# Supplementary material for: Synergistic Cytokine Production by ATP and PGE2 via P2X4 and EP3 Receptors in Mouse Bone-Marrow-Derived Mast Cells
Source: Cells. 2022 Feb 10;11(4):616. doi: 10.3390/cells11040616 (PMC8870111; doi:10.3390/cells11040616)
Supplement: Supplementary file 1 [file cells-11-00616-s001.zip › cells-1552650-supplementary.pdf]

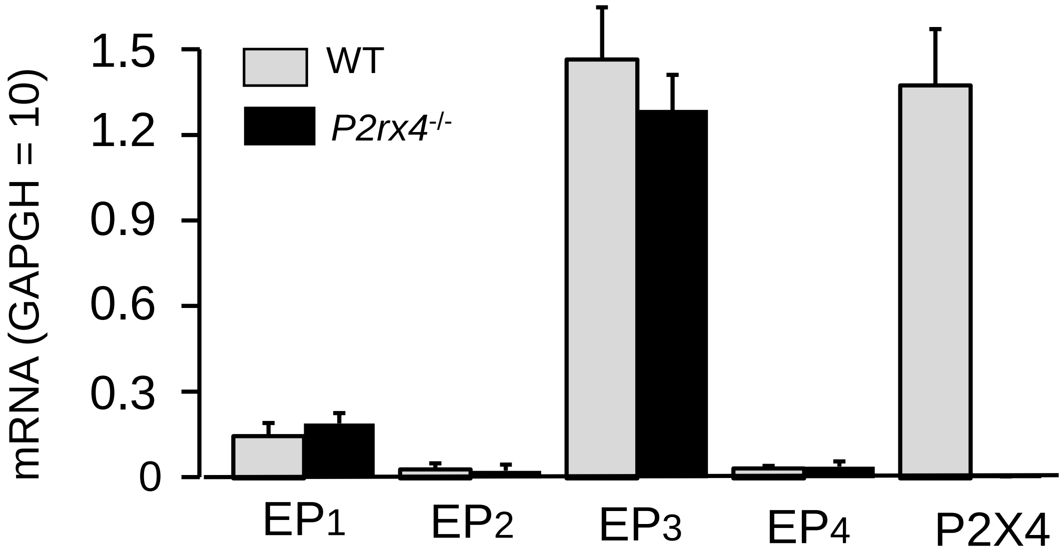

Supplemental Figure S1

Expression of mRNAs for EP receptor subtypes in BMMC obtained from wild type (WT) and P2X4 receptor deficient (*P2rx4*$^{-/-}$) mice. EP_1,EP_2, EP_3, EP_4 and P2X4 receptor mRNA expression were examined by quantitative RT-PCR. Data were normalized to GAPDH mRNA levels. Values are shown as mean ± SEM (n = 3).

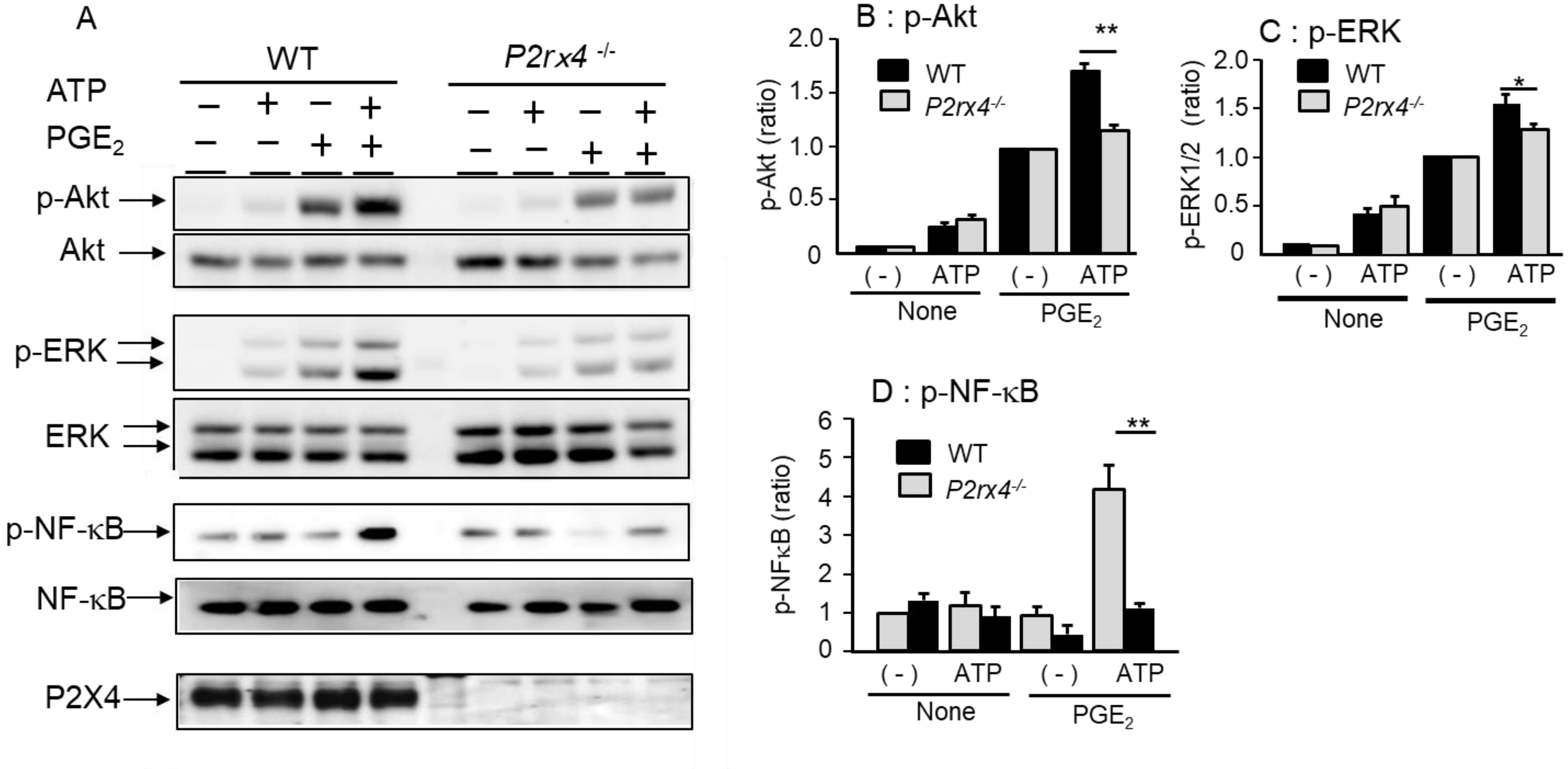

Supplemental Figure S2

Effects of co-stimulation of ATP and PGE_2 on the phosphorylation of ERK1/2, Akt, and NF-κB. (A) BMMCs prepared from WT (left) and *P2rx4*$^{-/-}$ mice (right) were stimulated with vehicle or PGE_2 (0.1 μM) with or without ATP (100 μM) for 5 min to evaluate phospho-NF-κB or for 20 min to examine phospho-Akt and phospho-ERK1/2. Cell lysates were subjected to western blot analysis. P2X4R protein was examined with anti-P2X4R antibody. (B,C,D) The densitometry value of protein bands of phospho-Akt (B), phospho-ERK 1/2 (C) are shown as relative intensity as 1 for the results obtained with PGE_2 alone. The densitometry values of the protein band of phospho-NF-kB (D) were shown as relative intensity as 1 for the results obtained in unstimulated cells (none). Data are shown as mean ± SEM (n = 3). *$P < 0.05$, **$P < 0.01$.
